# Supplementary material for: Ultrafast THz probing of nonlocal orbital current in transverse multilayer metallic heterostructures
Source: Nat Commun. 2023 Dec 11;14:8185. doi: 10.1038/s41467-023-43956-y (PMC10713980; doi:10.1038/s41467-023-43956-y)
Supplement: Supplementary file 1 — Supplementary Information [file 41467_2023_43956_MOESM1_ESM.pdf]

Supplementary Information

Ultrafast THz probing of nonlocal orbital current in transverse multilayer metallic heterostructures

Sandeep Kumar and Sunil Kumar*

Femtosecond Spectroscopy and Nonlinear Photonics Laboratory,
Department of Physics, Indian Institute of Technology Delhi, New Delhi 110016, India
**Email: kumarsunil@physics.iitd.ac.in*

S1. Structural characterization of the samples

S2. Secondary ion mass spectroscopy (SIMS) measurements

S3. M-H measurements

S4. M-T measurements

S5. Temperature-dependent THz time-domain spectroscopy setup

S6. THz spectral bandwidth

S7. Temperature-dependent longitudinal electrical resistivity measurements

S8. ISHE mediated THz emission from CoFeB/Ta bilayer

S9. Orbital transport in CoFeB/W(1)/Ta

S10. Effective spin-orbital Hall resistivity from the THz electric field strength

S11. THz amplitude dependency on the ultrafast excitation fluence

S12. THz emission from the FM layer alone

S13. Nb thickness dependent THz emission from NiFe/Nb bilayer

S14. THz emission from the CoFeB/W bilayer

S15. Temperature-dependent optical transmission of the substrate

S16. One-dimensional spin-orbit drift diffusion model for analyzing the enhancement in the orbital current with the thickness of the W-insertion layer

S1. Structural characterization of the samples

Figure S1 presents the results from X-ray diffraction (XRD), X-ray reflectivity (XRR), and atomic force microscopy (AFM) for determining the crystalline phase, thickness, roughness, and other characteristics of the thin film samples. The X-ray measurements were carried out using PANalytical X'Pert diffractometer with a Cu-K_α source. The XRD pattern of NiFe/Nb bilayer is shown in Fig. S1(a), where strong diffraction peaks at 38.6^0, 48.4^0, 55.4^0, and $69.3^{0,}$ corresponding to the crystalline planes of NiFe and Nb are marked. These results show that the Nb film is grown in the body centered cubic form while NiFe is grown in the face centered cubic form. To achieve growth of the W and Ta films in their low resistive α-phase, optimized growth rates of 0.05 nm/sec(Ta) and 0.03 nm/sec(W) were used while deposition power was kept constant[1, 2] at 50 W. A balanced growth rate along with the optimized deposition power is necessary to obtain the desired phase formation[3, 4]. Although all the samples were grown using the respective optimized growth rates for all the materials, the film/layer thickness and roughness were further confirmed by performing XRR. Figure S1(b) shows the XRR profile of NiFe/Nb bilayer sample. The experimental data in green open circles is fitted using GenX software to obtain information about film thickness and roughness. The AFM image as showin in Fig. S1(c) further confirms the surface morphology and roughness of the same sample. Root mean squared roughness extracted from the AFM result is found to be consistent with the one obtained from the XRR measurements. A small surface roughness incontrovertibly indicates very smooth sample quality. Other samples, such as CoFeB/W(1)/Ta (Fig. S1(d)), CoFeB/W(2)/Ta (Fig. S1(e)), were also examined in the similar fashion by utilizing XRR for their thickness and roughnesses in addition to the complementary AFM (Fig. S1(f)). Again, a lower value of surface and interfacial roughness confirms the very good quality of our prepared thin film samples.

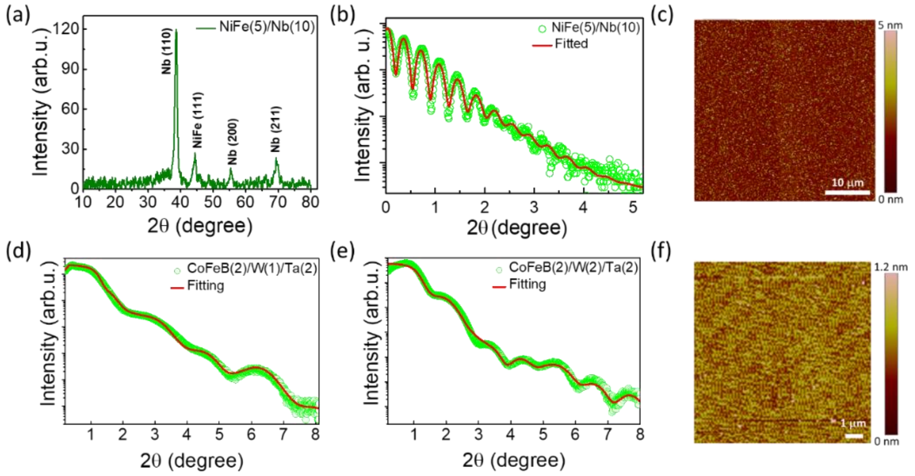

Figure S1. (a) XRD, (b) XRR, (c) AFM results on the NiFe/Nb bilayer sample. The crystallographic planes corresponding to various XRD peaks have been marked. XRR spectra of (d) CoFeB/W(1)/Ta and (e) CoFeB/W(2)/Ta trilayer samples. Numbers inside the small parentheses indicate film thickness in nm. (f) AMF image of CoFeB/W(2)/Ta sample. The solid red curves in (b,d,e) are fits to the experimental data for obtaining film thickness and surface roughness.

S2. Secondary ion mass spectroscopy (SIMS) measurements

To reconfirm the elemental composition over the thickness as well as the interface quality, we have performed secondary ion mass spectroscopy (SIMS) on a few selected samples, including CoFeB(2)/Ta(2), CoFeB(2)/W(2)/Ta(2) and NiFe(5)/Nb(10). The number inside the small parenthesis represents the film thickness in nm. A O_2^+ ion source of energy 0.5 keV was used for the depth profile measurements. The results are shown in Fig. S2, where the yield, referred as normalized intensity, is plotted with respect to the sample thickness from bottom to the top order of the stack. It is evident from the depth profiles that the elemental compositions of a given layer covered a range of thicknesses, and this thickness is consistent with that determined from the structural measurements made in section S1. For example, in the case of NiFe(5)/Nb(10) (Fig. S2(a)), the Ni and Fe elements span a thickness of 5 nm, while the Nb starts after this and ends at a thickness of ~15 nm. By comparing the results in Figs. S2(b) and S2(c), thickness of 2 nm is confirmed for the W-insertion layer.

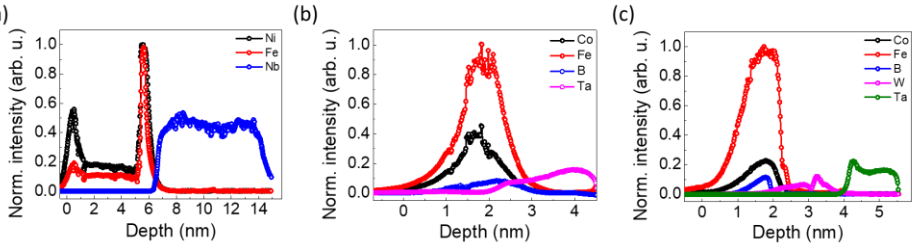

Figure S2. Secondary ion mass spectroscopy depth profiles of various elements in the thin film heterostructures of (a) NiFe(5)/Nb(10), (b) CoFeB(2)/Ta(2), and (c) CoFeB(2)/W(2)/Ta(2). The numbers inside small parentheses represent the corresponding film thickness in the nm.

S3. M-H measurements

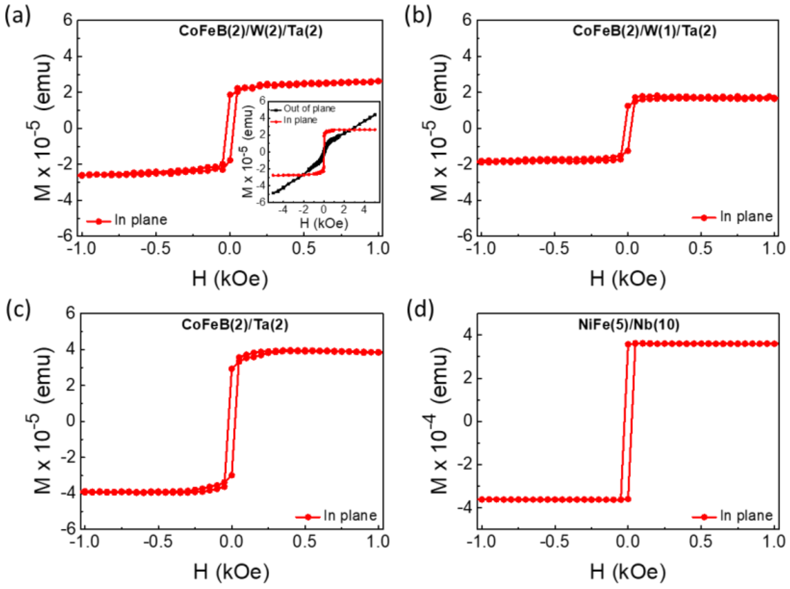

Figure S3. Magnetic-hysteresis loops from in-plane magnetic measurements using a vibrating sample magnetometer for (a) CoFeB(2)/W(2)/Ta(2), (b) CoFeB(2)/W(1)/Ta(2), (c) CoFeB(2)/Ta(2), and NiFe(5)/Nb(10) spintronic heterostructures. The numbers inside the small parentheses represent thicknesses or corresponding films in nm. Inset in (a) shows an out-of-plane M-H measurement for a comparison with the in-plane M-H measurement on the CoFeB(2)/W(2)/Ta(2) sample.

Vibrating sample magnetometer magnetic-hysteresis (M-H) measurements were performed using a quantum design magnetic properties measurements system. Figures S3(a) to (d) show the in-plane M-H loops of CoFeB(2)/W(2)/Ta(2), CoFeB(2)/W(1)/Ta(2), CoFeB(2)/Ta(2), and NiFe(5)/Nb(10) heterostructures, respectively. The number inside small parenthesis represents respective film thickness in the nm. Although a clear in-plane magnetic anisotropy is evident for all the samples but we have also performed the out-of-plane measurement for a few for completement and comparison. The in-plane and out-of-plane M-H loops for one of the samples, i.e., CoFeB(2)/W(2)/Ta(2) are shown in the inset of Fig. S3(a). The nonsaturating behavior in the case of out-of-plane M-H in the given range of field confirms the absence of any out-of-plane anisotropy. Usually, a perpendicular magnetic anisotropy occurs in the case of W-interlayer having high resistivity[5]. However, we do not find any change due to the W-insertion layer in M-H response of the CoFeB/W/Ta sample.

S4. M-T measurements

We performed field-cooled (FC) and zero field-cooled (ZFC) magnetization measurements as a function of the sample temperature after cooling the heterostructures from 300 K to 10 K in an applied in-plane magnetic field of 600 (100) Oe (FC) and 0 Oe (ZFC). The 600 Oe and 100 Oe FC values are corresponding to the CoFeB/W/Ta and NiFe/Nb samples, respectively. FC and ZFC data were also collected while warming the heterostructures in a field of 600 Oe and 100 Oe. Figure S4 summarizes the above results obtained for the saturation magnetization (M_S) as a function of the sample temperature. It is evident from the figures that the temperature-dependent behaviour of the magnetization remains the same throughout the temperature range. Therefore, the influence of any temperature-

dependent change of the magnetic properties can be safely avoided in our experiments for THz emission from them.

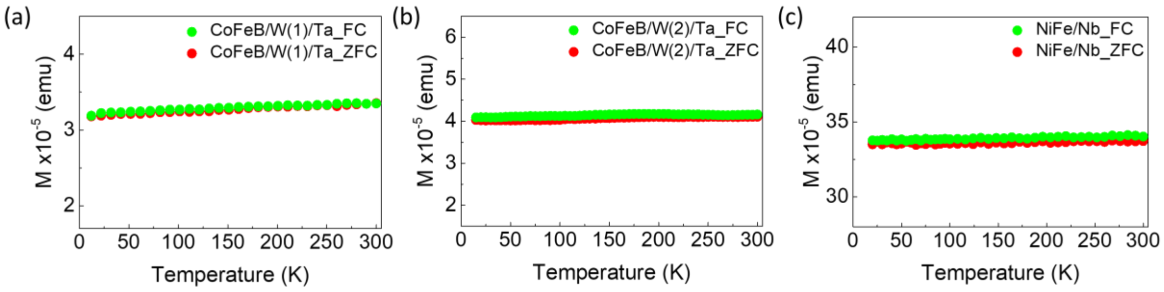

Figure S4. Saturation magnetization (M_S) as a function of varying sample temperature for (a) CoFeB/W(1)/Ta, (b) CoFeB/W(2)/Ta, and (c) NiFe/Nb samples obtained during field cooling and zero field cooling measurements.

S5. Temperature-dependent THz time-domain spectroscopy setup

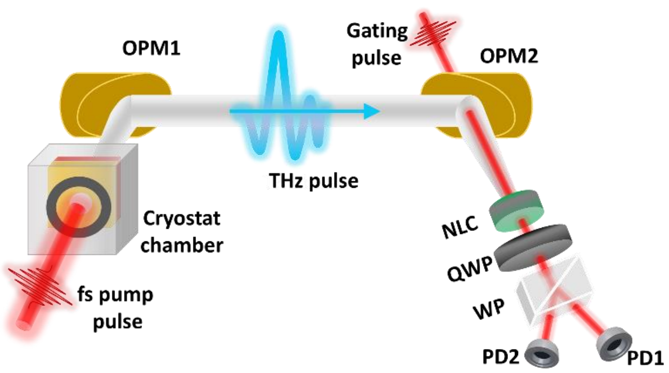

Figure S5. Cryogenically combined ultrafast optical-THz setup for temperature-dependent THz emission time-domain measurements. OPM: Off-axis parabolic mirror, QWP: Quarter-wave plate, NLC: Nonlinear crystal, WP: Wollaston prism, PD: Photodiode.

S6. THz spectral bandwidth

Figure S6 presents typical fast-Fourier transformation (FFT) spectra of the broadband THz pulses emitted from our spintronic heterostrutures, here shown for the NiFe/Nb sample for its two different optical excitation configurations. In the first configuration, the sample is excited from the substrate side, while in the other, the optical excitation is done from the film sample side.

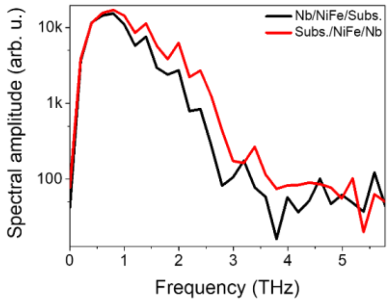

Figure S6. Typical Fast Fourier Transform (FFT) spectra of the THz emission time-domain signals generated from NiFe/Nb bilayer by exciting with the femtosecond pulase at 800 nm either from the substrate or the opposite side.

S7. Temperature-dependent longitudinal electrical resistivity measurements

In Figure S7, we have presented results for temperature-dependent longitudinal resistivity by using four-point van der Pauw method for different material layers and their combinations/heterostructures. Parallel resistive model[1, 6] was used to determine the resistivity of the W/Ta bilayer as shown in Figure S7(c).

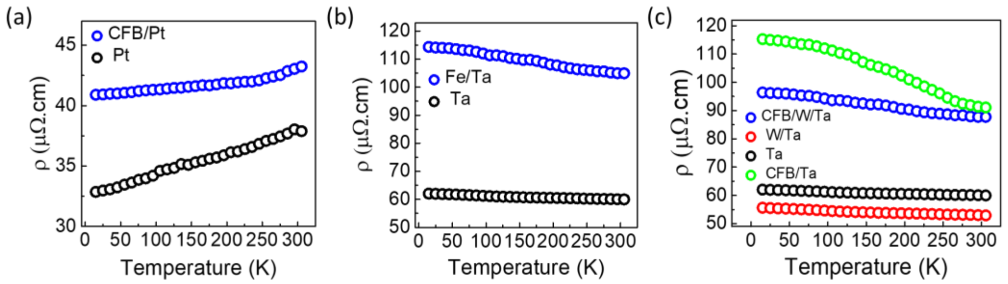

Figure S7. Temperature-dependent longitudinal resistivity (ρ or ρ_{xx}) measured by four-point van der Pauw method for (a) CoFeB/Pt and Pt, (b) Fe/Ta and Ta, (c) CoFeB/W/Ta, CoFeB/Ta, W/Ta, and Ta thin film samples.

S8. ISHE mediated THz emission from CoFeB/Ta bilayer

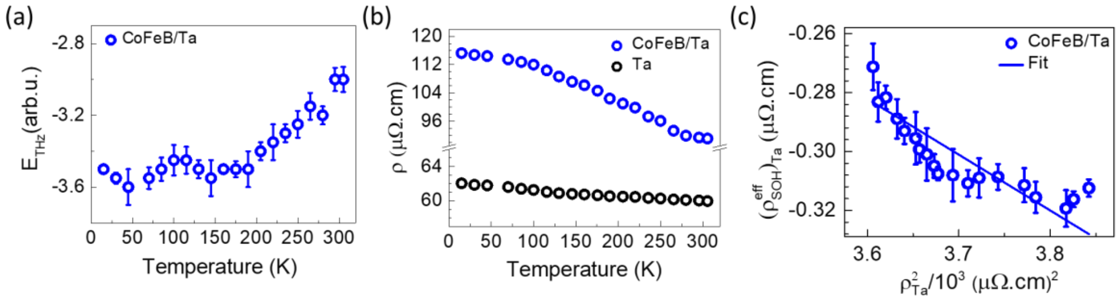

Figure S8. (a) Peak-to-peak THz signal variation with the varying sample temperature for CoFeB/Ta heterostructure. The error bars at each temperature correspond to the largest absolute deviation of the peak-to-peak THz amplitude from the mean of three measurements. (b) Longitudinal resistivities (ρ) as a function of temperature measured by the four-point van der Pauw method. (c) Effective spin-orbital Hall resistivity vs squared longitudinal resistivity plot for Ta.

Temperature dependent behaviors of the emitted THz signal from CoFeB/Ta bilayer and its longitudinal resistivity are presented in Figs. S8(a) and S8(b), respectively. Using these resuls in Figs. S8(a) and (b), we determined the temperature-dependent behavior of the effective spin-orbital Hall resistivity vs squared longitudinal resistivity of Ta as shown in Fig. S8(c). Solid line in Fig. S8(c) is a linear fit to the data using Eq. (4) of the main manuscript. We note that Ta possesses a negatively valued spin Hall conductivity and a positively valued orbital Hall conductivity. The negative slope of the fitted curved in Fig. S8(c) and its value is consistent with the negative value of spin Hall conductivity in Ta signifying that ISHE is the source of THz radiation from CoFeB/Ta bilayer.

S9. Orbital transport in CoFeB/W(1)/Ta

We also performed temperature-dependent THz emission and subsequent extraction of the effective intrinsic spin-orbit Hall conductivity of CoFeB/W/Ta trilayer having 1 nm thick W-insertion layer. Figure S9(a) shows the peak-to-peak amplitude of THz time-domain signal vs the sample temperature. The longitudinal resistivities of CoFeB/W(1)/Ta and W/Ta as a function of temperature are provided in Fig. S9(b). The information from Figs. S9(a) and S9(b) are used to extract the results in Fig. S9(c), where the effective spin-orbital Hall resistivity ρ_{SH}^{eff} has been plotted as a function of squared longitudinal resistivity of W/Ta. In this figure, solid curve is linear fit to the data using Eq. 4 of the main manuscript. From the negative slope of the linear fit, we have determined the intrinsic spin Hall conductivity value of $(\sigma_{SH}^{int})^{eff} = -1250\ (\hbar/e)\ \Omega^{-1} cm^{-1}$.

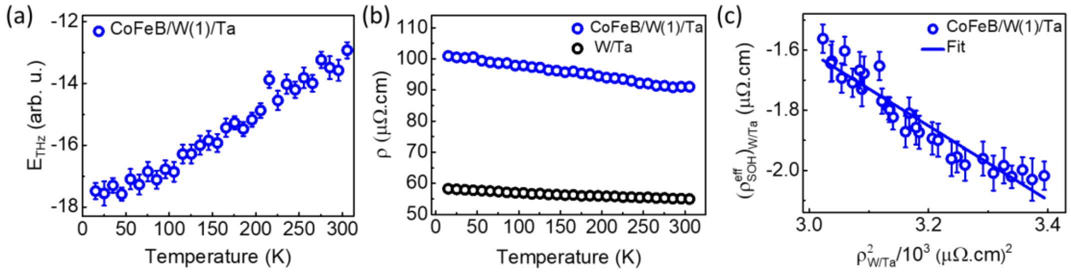

Figure S9. (a) Peak-to-peak THz signal amplitude variation with respect to the sample temperature for CoFeB/W(1)/Ta. The error bars at each temperature correspond to the largest absolute deviation of the peak-to-peak

THz amplitude from the mean of three measurements. (b) Temperature-dependent longitudinal resistivity of CoFeB/W/Ta and W/Ta samples. (c) Effective spin-orbital Hall resistivity ρ_{SH}^{eff} as a function of the squared longitudinal resistivity of W/Ta.

S10. Effective spin-orbital Hall resistivity from the THz electric field strength

The THz electric field strength (E_{THz}) from the measured electro-optic signal can be estimated using the standard procedure[1, 7, 8] that is discussed briefly here. Considering the fact that the spintronic emitters share almost a similar emission efficiency,[9] the THz electric field from the measured differential intensity ($\Delta I/I$) by electro-optic sampling in the ZnTe nonlinear crystal or our expeirmental setup, is estimated using the following expression.

$$E_{THz}\left(\frac{V}{cm}\right) = \left(\frac{\Delta I}{I}\right)\frac{2c}{\omega n^3 r_{41} L}.\left(\frac{1}{\cos\alpha\ \sin 2\beta + 2\sin\alpha\ \cos 2\beta}\right) \quad \text{(S1)},$$

where, the c = 3x10^8 m/s, $\omega = 2\pi\nu = 2\pi c/800$nm, L = 0.5mm, n = 2.85[10], r_{41} = 3.9x10^{-12} m/V^7, $\alpha = 90^0$ and $\beta = 180^0$ in our experiments for the THz electric field at the ZnTe crystal.

For the THz emission by ISHE in FM/NM-based heterostructures, the magnitude of the corresponding THz electric field depends on various parameters via the relation,[11, 12]

$$E(\omega)_{THz} = \frac{e.J_S(\omega).\theta_{SH}.\lambda_S}{(1+n)/Z_0 + \int_0^d \sigma(\omega,z)dz} \quad \text{(S2)}$$

Here, n, Z_0, θ_{SH}, e, J_S, λ_S and d are substrate refractive index, vacuum impedance, spin Hall angle, elementary charge, spin current density, spin relaxation length and thickness of the heterostructure, respectively. The electronic charge is included in the above formula because J_S and J_C (charge current density) are used in the units of $\hbar$ and e, respectively. The shunt conductance $(1+n)/Z_0$ is much lower than conductance of the heterostructures $\int_0^d \sigma(\omega,\ z)dz$, therefore the former can be neglected in the estimation. Using $\int_0^d \sigma(\omega,\ z)dz = d/\rho_{FM/NM}$, Eq. (S2) changes to

$$E(\omega)_{THz} = \frac{e.J_S(\omega).\theta_{SH}.\lambda_S}{(d/\rho_{FM/NM})} \quad \text{(S3)},$$

where, a fact has been used that the conductivity depends only weakly on the THz frequencies[11, 13] and hence can be approximated to a constant. Ignoring any frequency-dependence of the other quantities, the corresponding relation in the time-domain becomes,

$$E(t)_{THz} = \frac{e.J_S(t).\theta_{SH}.\lambda_S}{(d/\rho_{FM/NM})} \quad \text{(S4)}$$

Therefore, the instantaneous value of the spin-current generated by ultrafast excitation can be quantified directly from the maximum of the time-domain THz signal. Also, the spin current is generated from FM layer is nearly temperature insensitive since all the measurements were performed well below the Curie temperature, as confirmed by M-T measurements shown in Section S4. Without loss of generality, the peak-to-peak value of the experimentally measured THz field has been considered for the latter in all our analysis. The spin Hall angle θ_{SH} is usually defined[14] as $\theta_{SH} = \rho_{SH}^{NM}/\rho_{xx}^{NM}$, where, ρ_{SH}^{NM} and ρ_{xx}^{NM} represent the spin Hall resistivity and longitudinal resistivity, respectively, of the NM layer. With all the above, a relationship between the spin Hall resistivity and the maximum of the measured THz field can be obtained as

$$\rho_{SH}^{NM} = E_{THz}\left(\frac{\rho_{xx}^{NM}}{\rho_{FM/NM}}\right)\left(\frac{d}{\lambda_S}\right)\frac{1}{e.J_S} \quad \text{(S5)}$$

For the case of THz emission by IOHE and ISHE, where both the spin current to charge current and orbital current to charge current conversions take place simultaneously, the above relation needs to be modified. IOHE becomes relevant if spin-orbit conversion takes place in the material layer via the spin-orbit correlation factor, η_{L-S}. Hence, for this case, the above relation gets modified to the following one,

$$E_{THz} = \frac{e.J_S.\lambda_{LS}}{(d/\rho_{FM/NM})}\left(\frac{\rho_{SH}^{NM}}{\rho_{xx}^{NM}} + \frac{\rho_{OH}^{NM}}{\rho_{xx}^{NM}}.\eta_{L-S}\right) \quad \text{(S6)}$$

Notice that, J_S is retained in the above relation because it represents the maximum instantaneous spin current produced in the FM layer by the ultrafast optical excitation, however, the spin relaxation length has been substituted by the effective spin-orbit diffusion length,[15] $\lambda_{LS} = \sqrt{\lambda_L \lambda_S}$ (λ_L and λ_S being the orbital and spin diffusion lengths) and θ_{SH} has been substituted by an effective spin-orbit Hall angle[16], $\theta_{SOH}^{eff} = \left(\frac{\rho_{SH}^{NM}}{\rho_{xx}^{NM}} + \frac{\rho_{OH}^{NM}}{\rho_{xx}^{NM}}.\eta_{L-S}\right)$ to take into account the orbital Hall resistivity, ρ_{OH}^{NM} of the NM layer. The θ_{SOH}^{eff} effectively takes care of the overall effect of spin and orbital currents, and their interconversion on the ultimate charge current and hence THz radiation

produced.[15, 16] Equation (S6) is strictly valid for unit interfacial transparency,[16, 17] which can be rearranged to obtain a relation between the effective spin-orbit Hall resistivity, ρ_{SOH}^{eff} and the measured THz signal as

$$\rho_{SOH}^{eff} = (\rho_{SH}^{NM} + \rho_{OH}^{NM} . \eta_{L-S}) = E_{THz} \left(\frac{\rho_{xx}^{NM}}{\rho_{FM/NM}} \right) \left(\frac{d}{\lambda_{LS}} \right) \frac{1}{eJ_S} \tag{S7}$$

Now, at each sample temperature T, the value of the experimentally measured THz signal $E_{THz}(T)$ in V/cm from Eq. (S1) can be used to calculate the effective spin-orbit Hall resistivity $\rho_{SOH}^{eff}(T)$ in the units of $\mu\Omega.cm$ using Eq. (S7) for the complete temperature-dependent analysis.

S11. THz amplitude dependency on the ultrafast excitation fluence

Ultrafast excitation fluence dependence of the THz emission efficiecy of various heterostructures at the room tempreature was also evaluaed. Some of the representative results are presented in Fig. S10. It can be seen that the THz signal magnitude follows nearly linear dependence on the excitation fluences up to a large fluence value for all the samples. However, a slight deviation from the linear behaviour is observed for the NiFe/Nb and CoFeB/W/Ta heterostructures. We note that both types of these heterostructures emit THz radiation due to IOHE in the NM layer. A similar weakly saturating behaviour with respect to the excitation fluence has also been reported very recently by another group[18] in which case also, the THz radiation generation was majorly attributed to ultrafast IOHE in the NM layer.

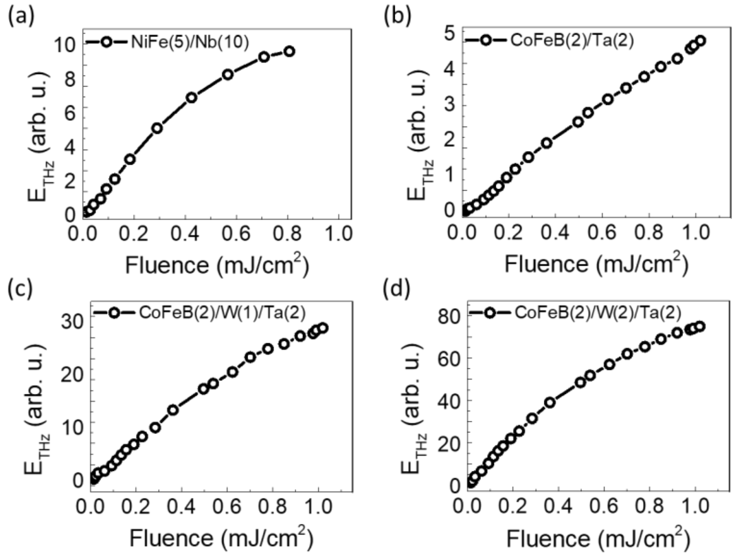

Figure S10. Peak to peak THz amplitude with the varying ultrafast pump fluence for (a) NiFe(5)/Nb(10), (b) CoFeB(2)/Ta(2), (c) CoFeB(2)/W(1)/Ta(2), and (d) CoFeB(2)/W(2)/Ta(2) heterostructures. The integers inside small parentheses represent thickness of respective layers in nm.

S12. THz emission from the FM layer alone

We have also recorded a weak THz emission from the FM layers alone. The corresponding results for the NiFe and CoFeB are presented in Fig. S11. Figure S11(a) shows the THz time-doamin signal emitted from bare $Ni_{0.9}Fe_{0.1}$ sample. As evident from the figure, the THz signal polarity remains the same irrespective of the sample excitation geometry. The terms, back side and front side in the figure are designated for the substrate side and film side optical pumping, respectively. The THz emission from a different FM material layers has also been demonstrated in various reports in the literature.[19-22] In all such cases, the origin for the weak THz emission can be attributed mainly to either the anomalous Hall effect (AHE)[19, 21, 22] or ultrafast demagnetization[23-25] (UDM). Both of these mechanisms have different origins and follow different characteristics, which can be distinguished qualitatively by analyzing THz polarity behavior with respect to the sample excitation geometry. The polarity of the THz waveform is reversed when the sample is flipped, a phenomenon that can be attributed to the AHE[19, 21, 22] resulting from a change in the direction of the net backflow current. However, in the case of ultrafast demagnetization[23-25] (UDM), the THz waveform polarities remain the same upon sample flipping due to the fact that the magnetization dynamics is insensitive to the sample excitation direction geometry. Our results on NiFe(5) are found to be well aligned with the latter scenario as apparent from Fig. S11(a). We have compared the THz signals from bare NiFe and the NiFe/Nb bilayer in Figure S11(b). A significantly large difference between the THz signal magnitudes from the two samples clearly indicates only weak THz emission due to UDM in the NiFe layer. Furthermore, the

consistency of our observation with the UDM in NiFe rules out any possibilities for THz emission from NiFe(5nm)/Nb(10nm) sample due to other mechanisms involving AHE[19, 21, 22] and structural inversion symmetry.[26] Similar outcomes are also observed in the case of bare CoFeB sample (see Fig. S11(c)) to reiterate that very weak THz emission from CoFeB layer is not due to AHE.

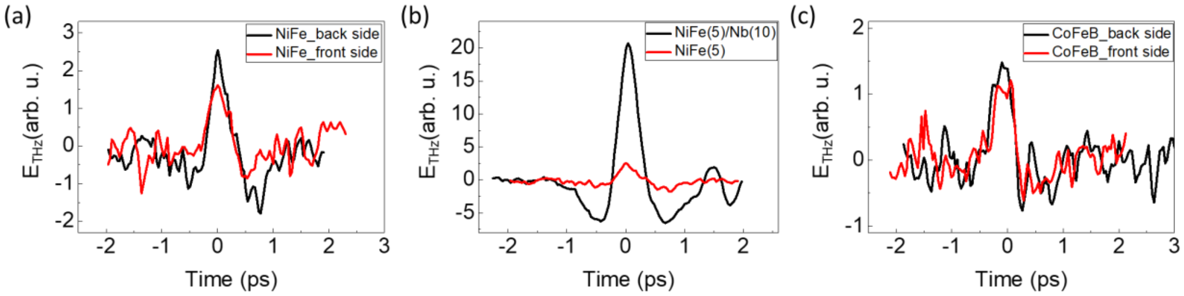

Figure S11. Time-domain THz waveforms generated from (a) bare NiFe layer under front- and back-side optical pumping, (b) NiFe and NiFe/Nb samples, and (c) bare CoFeB layer under front- and back-side optical pumping. The integers inside small parentheses are thicknesses of the respective layers in nm.

S13. Nb thickness dependent THz emission from NiFe/Nb bilayer

Figure S12 presents results for the THz emission from NiFe/Nb samples and to compare the THz signal magnitude with respect to the thickness of the Nb layer. Figure S12(a) presents the raw data and explicit Nb-thickness dependence of the THz signal magnitude that is normalized with both optical absorbance and THz impedance is shown in Figure S12(b). The latter is calculated[18, 27, 28] by using transmitted THz signal amplitudes from the samples and reference (see Fig. S12 (c)), which are obtained by performing the time-domain THz transmission measurements with an ultrafast air-plasma based THz sources. For these measurements on NiFe/Nb bilayers of varying Nb thickness, the NiFe layer thickness was kept the same at 5 nm for all. We can see that the THz signal polarity is same for all the samples and its magnitude decreases strongly for higher thicknesses of the Nb-layer in NiFe/Nb bilayer. As brought out clearly in our main manuscript that IOHE is the source of THz radiation from the NiFe/Nb bilayer, we believe that our results on the Nb-thickness dependence of the THz emission (Fig. S12(b)) is a new value addition to the field. From Fig. S12(b), we also conclude that the orbital current diffusion length in Nb is of ~25 nm, that is in the same order as the spin current diffusion length in it at the room temperature.[29] A similar value persists at the low temperatures as well.

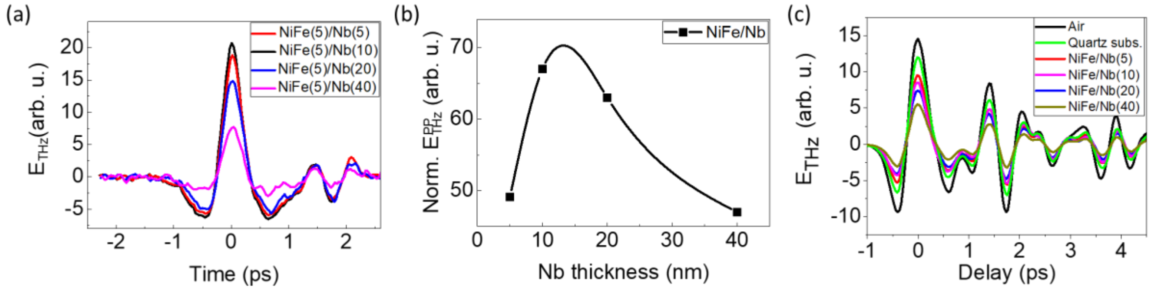

Figure S12. (a) Time-domain THz waveforms emitted from NiFe/Nb bilayer with the varying thickness of Nb layer. (b) Corresponding peak-to-peak THz signal amplitude variation with the Nb layer thcikness. (c) As recorded THz transmission signals from different NiFe/Nb samples of varying Nb thickness, including the reference signal in air and through the quartz substrate. The THz source for the measurements in (c) was a dual color ultrafast air plasma.

S14. THz emission from the CoFeB/W bilayer

Figure S13(a) presents results from the time-domain THz emission measurements on the CoFeB/W bilayers. A comparison in the THz signal generation from CoFeB(2)/W(2)/Ta(2), CoFeB(2)/W(2), and CoFeB(2)/Ta(2) samples is provided in Fig. S13(b). The number inside the small parenthesis is the respective film thickness in nm. From these results, certain conclusions can be made: (i) THz signal emitted from CoFeB/W(2nm) bilayer sample is ~2.5 times higher than that from CoFeB/Ta(2nm) whereas their polarities are same. (ii) ISHE is the origin of THz emission from both the CoFeB/W(2nm) and CoFeB/Ta(2nm) bilayers. Analogous to CoFeB/Ta(2nm) bilayer, ISHE is the origin of THz emission from CoFeB/W(2nm). The similar polarity and different THz amplitudes are quite consistent with the sign and magnitude of the spin Hall angles in W and Ta. (iii) THz signal from CoFeB/W bilayer decreases if the W layer thickness is increased from 2 to 3 nm. This clearly indicates that the spin diffusion

length in the W-layer, and hence it's optimum thickness for the THz emission via ISHE, is about 2-3 nm. Such a value of the spin diffusion length in W-layer matches well with the literature.[30] (iv) CoFeB/W(2)/Ta(2) trilayer sample emits ~10 times stronger signal than from CoFeB/Ta(2) bilayer and ~4 times higher THz signal as compared to that from the CoFeB/W(2) sample. For such a large difference in the THz emission efficiency of the trilayer as against the bilayer counterparts is argued to originate from W-insertion layer mediated enhanced orbital transport and its conversion to charge current in the Ta layer of the trilayer heterostructure.

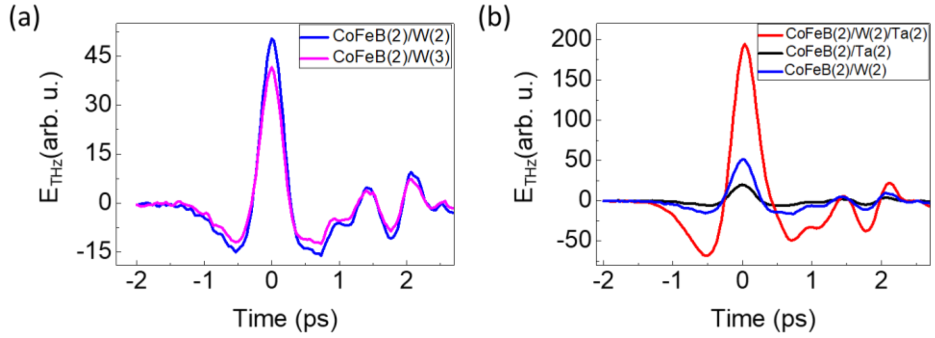

Figure S13. (a) Time-domain THz waveforms emitted from CoFeB(2)/W(2) and CoFeB(2)/W(3) bilayers. (b) Comparisons of THz signal amplitude for CoFeB(2)/W(2)/Ta(2), CoFeB(2)/W(2), and CoFeB(2)/Ta(2) samples. The numbers inside small parentheses represent thickness of the respective layer in nm.

S15. Temperature-dependent optical transmission of the substrate

Since we have performed all the THz emission measurements in the transmission mode, where the optical beam traverses the substrate first before reaching the film, it becomes important to check any temperature-dependent change in the optical transmission from the quartz substrate. Therefore, we have carried out optical transmission measurements on our bare quartz substrate under similar conditions of pump fluence and temperatures used for samples in our study. The corresponding result is shown in Fig. S14. There is hardly any change in the transmission, and it is less than a percent while traversing from the lowest temperature to the room temperature. We measured a negligible (<2%) change in the THz emission efficiency from our samples corresponding to the variation in the excitation power mentioned above. Such an insignificant excitation power variation does not affect the large temperature dependence of the THz signal as measured from our samples.

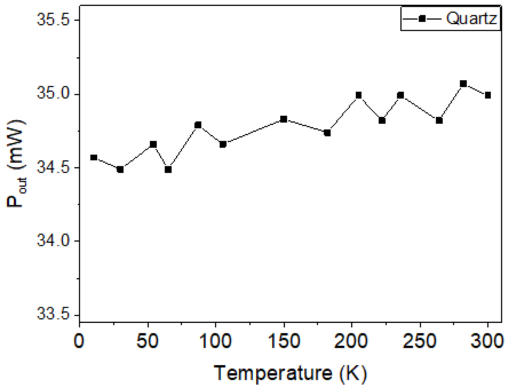

Figure S14. Temperature-dependent optical transmission at 800 nm from the substrate.

S16. One-dimensional spin-orbit drift diffusion model for analyzing the enhancement in the orbital current with the thickness of the W-insertion layer

As shown in Fig. 4 (c) of our manuscript, the THz signal from CoFeB/W(t)/Ta trilayer heterostructure increases with the increasing thickness (t) of the W-insertion layer. This observation is consistent with the fact that heavy metal W possesses large negative valued spin-orbit correlation factor owing to which efficient spin-orbit conversion occurs in it. Long diffusion length for the orbital current and high orbital Hall conductivity in the adjacent Ta layer profuse in tandem to generate stronger THz signal via IOHE from CoFeB/W/Ta trilayer and efficiency increases with the varying thickness of W-insertion layer. Following the assertions of spin-orbit interconversions by Sala et al.,[15] and the associated coupled differential equations for the chemical potentials and current densities related to the spin and orbital degrees, the thickness dependent enhancement in the OHE or the

ultrafast IOHE in our case can be analyzed. Figure S15 presents the response of the orbital current as a function of the W-insertion layer calculated using the phenomenological model of Sala et al.[15] for the orbital current given by the relation,

$$J_L(z_{NM}) = -\left(\frac{\sigma_S \mp \frac{\sigma_L}{\lambda_{LS}^2 \gamma_2}}{1-\frac{\gamma_2}{\gamma_1}}\right)\frac{E}{2}\,\mathrm{Sech}^2\left(\frac{z_{NM}}{2\lambda_1}\right) - \left(\frac{\sigma_S \mp \frac{\sigma_L}{\lambda_{LS}^2 \gamma_1}}{1-\frac{\gamma_1}{\gamma_2}}\right)\frac{E}{2}\,\mathrm{Sech}^2\left(\frac{z_{NM}}{2\lambda_2}\right) + \sigma_L E \quad \text{(S8)}$$

Here, different parameters have meaning as given in the original paper,[15] E is the applied external field in typical OHE settings, z_{NM} is the thickness of the heavy metal layer and so on. In generating the qualitative result of Fig. S15, we have used the fact that $\sigma_S < 0$ and $\sigma_L > 0$ for the heavy metal W.[31, 32] Clearly, a larger thickness of the W-insertion layer supports larger spin-orbital conversion. Hence, stronger orbital current is injected into the adjacent Ta layer which possesses much stronger orbital Hall conductivity than the spin Hall conductivity, thereby, resulting into much stronger orbit-charge conversion via IOHE and hence stronger THz emission from the trilayer with thicker W-insertion layer. We believe that our extensive temperature-dependent experiments for the THz emission via IOHE in CoFeB/W/Ta have much scope for further theoretical exploration and more experiments studies on such systems in future.

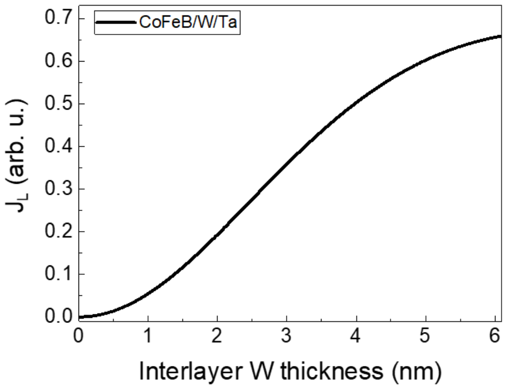

Figure S15. Increase in the orbital current with the increasing thickness of W-insertion layer in CoFeB/W/Ta trilayer.

References

1. Kumar, S.; Kumar, S., Large interfacial contribution to ultrafast THz emission by inverse spin Hall effect in CoFeB/Ta heterostructure. *iScience* **2022,** *25* (8), 104718.
2. Kumar, S.; Nivedan, A.; Singh, A.; Kumar, Y.; Malhotra, P.; Tondusson, M.; Freysz, E.; Kumar, S., Optical damage limit of efficient spintronic THz emitters. *iScience* **2021,** *24* (10), 103152.
3. Jhajhria, D.; Behera, N.; Pandya, D. K.; Chaudhary, S., Dependence of spin pumping in W/CoFeB heterostructures on the structural phase of tungsten. *Physical Review B* **2019,** *99* (1), 014430.
4. Kumar, A.; Bansal, R.; Chaudhary, S.; Muduli, P. K., Large spin current generation by the spin Hall effect in mixed crystalline phase Ta thin films. *Physical Review B* **2018,** *98* (10), 104403.
5. Li, S. K.; Zhao, X. T.; Liu, W.; Wang, T. T.; Zhao, X. G.; Zhang, Z. D., Enhanced spin-orbit torques and perpendicular magnetic anisotropy in CoFeB/MgO structures with Ta/W bilayer. *AIP Advances* **2018,** *8* (6), 065007.
6. Chen, Y.-Y.; Juang, J.-Y., Finite element analysis and equivalent parallel-resistance model for conductive multilayer thin films. *Measurement Science and Technology* **2016,** *27* (7), 074006.
7. Planken, P. C. M.; Nienhuys, H.-K.; Bakker, H. J.; Wenckebach, T., Measurement and calculation of the orientation dependence of terahertz pulse detection in ZnTe. *J. Opt. Soc. Am. B* **2001,** *18* (3), 313-317.
8. Cheng, L.; Wang, X.; Yang, W.; Chai, J.; Yang, M.; Chen, M.; Wu, Y.; Chen, X.; Chi, D.; Goh, K. E. J.; Zhu, J.-X.; Sun, H.; Wang, S.; Song, J. C. W.; Battiato, M.; Yang, H.; Chia, E. E. M., Far out-of-equilibrium spin populations trigger giant spin injection into atomically thin MoS2. *Nature Physics* **2019,** *15* (4), 347-351.
9. Yang, D.; Liang, J.; Zhou, C.; Sun, L.; Zheng, R.; Luo, S.; Wu, Y.; Qi, J., Powerful and Tunable THz Emitters Based on the Fe/Pt Magnetic Heterostructure. *Advanced Optical Materials* **2016,** *4* (12), 1944-1949.
10. Li, H. H., Refractive Index of ZnS, ZnSe, and ZnTe and Its Wavelength and Temperature Derivatives. *Journal of Physical and Chemical Reference Data* **1984,** *13* (1), 103-150.
11. Seifert, T. S.; Tran, N. M.; Gueckstock, O.; Rouzegar, S. M.; Nadvornik, L.; Jaiswal, S.; Jakob, G.; Temnov, V. V.; Münzenberg, M.; Wolf, M.; Kläui, M.; Kampfrath, T., Terahertz spectroscopy for all-optical spintronic characterization of the spin-Hall-effect metals Pt, W and Cu80Ir20. *Journal of Physics D: Applied Physics* **2018,** *51* (36), 364003.
12. Seifert, T.; Jaiswal, S.; Martens, U.; Hannegan, J.; Braun, L.; Maldonado, P.; Freimuth, F.; Kronenberg, A.; Henrizi, J.; Radu, I.; Beaurepaire, E.; Mokrousov, Y.; Oppeneer, P. M.; Jourdan, M.; Jakob, G.; Turchinovich, D.; Hayden, L. M.; Wolf, M.; Münzenberg, M.; Kläui, M.; Kampfrath, T., Efficient metallic spintronic emitters of ultrabroadband terahertz radiation. *Nature Photonics* **2016,** *10*, 483.
13. Seifert, T.; Martens, U.; Günther, S.; Schoen, M. A. W.; Radu, F.; Chen, X. Z.; Lucas, I.; Ramos, R.; Aguirre, M. H.; Algarabel, P. A.; Anadón, A.; Körner, H. S.; Walowski, J.; Back, C.; Ibarra, M. R.; Morellón, L.; Saitoh, E.; Wolf, M.; Song, C.; Uchida, K.; Münzenberg, M.; Radu, I.; Kampfrath, T., Terahertz Spin Currents and Inverse Spin Hall Effect in Thin-Film Heterostructures Containing Complex Magnetic Compounds. *SPIN* **2017,** *07* (03), 1740010.
14. Sagasta, E.; Omori, Y.; Isasa, M.; Gradhand, M.; Hueso, L. E.; Niimi, Y.; Otani, Y.; Casanova, F., Tuning the spin Hall effect of Pt from the moderately dirty to the superclean regime. *Physical Review B* **2016,** *94* (6), 060412.
15. Sala, G.; Gambardella, P., Giant orbital Hall effect and orbital-to-spin conversion in 3d, 5d, and 4f metallic heterostructures. *Physical Review Research* **2022,** *4* (3), 033037.
16. Lee, S.; Kang, M.-G.; Go, D.; Kim, D.; Kang, J.-H.; Lee, T.; Lee, G.-H.; Kang, J.; Lee, N. J.; Mokrousov, Y.; Kim, S.; Kim, K.-J.; Lee, K.-J.; Park, B.-G., Efficient conversion of orbital Hall current to spin current for spin-orbit torque switching. *Communications Physics* **2021,** *4* (1), 234.
17. Go, D.; Freimuth, F.; Hanke, J.-P.; Xue, F.; Gomonay, O.; Lee, K.-J.; Blügel, S.; Haney, P. M.; Lee, H.-W.; Mokrousov, Y., Theory of current-induced angular momentum transfer dynamics in spin-orbit coupled systems. *Physical Review Research* **2020,** *2* (3), 033401.
18. Seifert, T. S.; Go, D.; Hayashi, H.; Rouzegar, R.; Freimuth, F.; Ando, K.; Mokrousov, Y.; Kampfrath, T., Time-domain observation of ballistic orbital-angular-momentum currents with giant relaxation length in tungsten. *Nature Nanotechnology* **2023**.
19. Zhang, Q.; Luo, Z.; Li, H.; Yang, Y.; Zhang, X.; Wu, Y., Terahertz Emission from Anomalous Hall Effect in a Single-Layer Ferromagnet. *Physical Review Applied* **2019,** *12* (5), 054027.
20. Huang, L.; Lee, S.-H.; Kim, S.-D.; Shim, J.-H.; Shin, H. J.; Kim, S.; Park, J.; Park, S.-Y.; Choi, Y. S.; Kim, H.-J.; Hong, J.-I.; Kim, D. E.; Kim, D.-H., Universal field-tunable terahertz emission by ultrafast photoinduced demagnetization in Fe, Ni, and Co ferromagnetic films. *Scientific Reports* **2020,** *10* (1), 15843.
21. Liu, Y.; Cheng, H.; Xu, Y.; Vallobra, P.; Eimer, S.; Zhang, X.; Wu, X.; Nie, T.; Zhao, W., Separation of emission mechanisms in spintronic terahertz emitters. *Physical Review B* **2021,** *104* (6), 064419.
22. Mottamchetty, V.; Rani, P.; Brucas, R.; Rydberg, A.; Svedlindh, P.; Gupta, R., Direct evidence of terahertz emission arising from anomalous Hall effect. *Scientific Reports* **2023,** *13* (1), 5988.

23. Beaurepaire, E.; Turner, G. M.; Harrel, S. M.; Beard, M. C.; Bigot, J. Y.; Schmuttenmaer, C. A., Coherent terahertz emission from ferromagnetic films excited by femtosecond laser pulses. *Applied Physics Letters* **2004,** *84* (18), 3465-3467.
24. Beaurepaire, E.; Merle, J. C.; Daunois, A.; Bigot, J. Y., Ultrafast Spin Dynamics in Ferromagnetic Nickel. *Physical Review Letters* **1996,** *76* (22), 4250-4253.
25. Zhang, W.; Maldonado, P.; Jin, Z.; Seifert, T. S.; Arabski, J.; Schmerber, G.; Beaurepaire, E.; Bonn, M.; Kampfrath, T.; Oppeneer, P. M.; Turchinovich, D., Ultrafast terahertz magnetometry. *Nature Communications* **2020,** *11* (1), 4247.
26. Rouzegar, R.; Brandt, L.; Nádvorník, L.; Reiss, D. A.; Chekhov, A. L.; Gueckstock, O.; In, C.; Wolf, M.; Seifert, T. S.; Brouwer, P. W.; Woltersdorf, G.; Kampfrath, T., Laser-induced terahertz spin transport in magnetic nanostructures arises from the same force as ultrafast demagnetization. *Physical Review B* **2022,** *106* (14), 144427.
27. Wang, P.; Feng, Z.; Yang, Y.; Zhang, D.; Liu, Q.; Xu, Z.; Jia, Z.; Wu, Y.; Yu, G.; Xu, X.; Jiang, Y., Inverse orbital Hall effect and orbitronic terahertz emission observed in the materials with weak spin-orbit coupling. *npj Quantum Materials* **2023,** *8* (1), 28.
28. Zhang, H.; Feng, Z.; Zhang, J.; Bai, H.; Yang, H.; Cai, J.; Zhao, W.; Tan, W.; Hu, F.; Shen, B.; Sun, J., Laser pulse induced efficient terahertz emission from Co/Al heterostructures. *Physical Review B* **2020,** *102* (2), 024435.
29. Jeon, K.-R.; Ciccarelli, C.; Kurebayashi, H.; Wunderlich, J.; Cohen, L. F.; Komori, S.; Robinson, J. W. A.; Blamire, M. G., Spin-Pumping-Induced Inverse Spin Hall Effect in Nb/Ni80Fe20 Bilayers and its Strong Decay Across the Superconducting Transition Temperature. *Physical Review Applied* **2018,** *10* (1), 014029.
30. Wang, T.-C.; Chen, T.-Y.; Wu, C.-T.; Yen, H.-W.; Pai, C.-F., Comparative study on spin-orbit torque efficiencies from W/ferromagnetic and W/ferrimagnetic heterostructures. *Physical Review Materials* **2018,** *2* (1), 014403.
31. Hayashi, H.; Jo, D.; Go, D.; Gao, T.; Haku, S.; Mokrousov, Y.; Lee, H.-W.; Ando, K., Observation of long-range orbital transport and giant orbital torque. *Communications Physics* **2023,** *6* (1), 32.
32. Salemi, L.; Oppeneer, P. M., First-principles theory of intrinsic spin and orbital Hall and Nernst effects in metallic monoatomic crystals. *Physical Review Materials* **2022,** *6* (9), 095001.
